# Darcy–Forchheimer couple stress hybrid nanofluids flow with variable fluid properties

**DOI:** 10.1038/s41598-021-98891-z

**Published:** 2021-10-04

**Authors:** Anwar Saeed, Poom Kumam, Taza Gul, Wajdi Alghamdi, Wiyada Kumam, Amir Khan

**Affiliations:** 1 Center of Excellence in Theoretical and Computational Science (TaCS-CoE), Faculty of Science, King Mongkut’s University of Technology Thonburi (KMUTT), 126 Pracha Uthit Rd., Bang Mod, Thung Khru, Bangkok, 10140 Thailand; 2 Department of Medical Research, China Medical University Hospital, China Medical University, Taichung, 40402 Taiwan; 3 Department of Mathematics, City University of Science and Information Technology, Peshawar, 25000 Pakistan; 4 Department of Information Technology, Faculty of Computing and Information Technology, King Abdulaziz University, Jeddah, 80261 Saudi Arabia; 5 Applied Mathematics for Science and Engineering Research Unit (AMSERU), Program in Applied Statistics, Department of Mathematics and Computer Science, Faculty of Science and Technology, Rajamangala University of Technology Thanyaburi, Thanyaburi, 12110 Pathumthani Thailand; 6 Department of Mathematics and Statistics, University Of Swat, Khyber Pakhtunkhwa, 19200 Pakistan

**Keywords:** Engineering, Mathematics and computing

## Abstract

The current study provides a detailed analysis of steady two-dimensional incompressible and electrically conducting magnetohydrodynamic flow of a couple stress hybrid nanofluid under the influence of Darcy–Forchheimer, viscous dissipation, joule heating, heat generation, chemical reaction, and variable viscosity. The system of partial differential equations of the current model (equation of motion, energy, and concentration) is converted into a system of ordinary differential equations by adopting the suitable similarity practice. Analytically, homotopy analysis method (HAM) is employed to solve the obtained set of equations. The impact of permeability, couple-stress and magnetic parameters on axial velocity, mean critical reflux condition and mean velocity on the channel walls are discussed in details. Computational effects show that the axial mean velocity at the boundary has an inverse relation with couple stress parameter while the permeability parameter has a direct relation with the magnetic parameter and vice versa. The enhancement in the temperature distribution evaluates the pH values and electric conductivity. Therefore, the $SWCNTs\,\,{\text{and}}\,\,MWCNTs$ hybrid nanofluids are used in this study for medication purpose.

## Introduction

Nanofluids contain significant application in the study of heat transfer enhancement. Different researchers showed that nanofluids has better heat transfer competence than traditional fluids, that’s why traditional fluids can be exchanged with nanofluids. Its higher thermal capability has diverted many researchers towards the study of nanofluids. This thermal conductivity property of nanofluids, distinguishes it from other fluids making it an important product for industrial sector including biomedicine, transportation, electronics, foods and nuclear reactors. The size of these nanoparticles is very small up to (1–100 nm) which enhanced the conductivity of the basic fluids upon addition. The structures of nanoparticles consist of metal oxide, carbide, nitride and carbon tubes (SWCNT-MWCNT) etc. The nanofluid has first introduced by Choi^1^, having applications ranging from manufacturing processes to industries. By applying the magnetic impact, the nanofluid with boundary layer flow towards the moving wedges has studied by Nadeem et al.^2^. Saleem et al.^3^ deliberated the heat transmission enhancement rate of different-shaped copper nanoparticles on the flat surface. Gul et al.^4^ have examined the fluid motion of a liquid film considering Reynolds model for the variable viscosity. The variable viscosity concept was used by the researchers in the blood flow containing nanomaterials. The valuable work of the researchers Huda et al.^5,6^, Ijaz et al.^7^, and Sheriff et al.^8^ related to the blood based nanofluids is more applicable. The CNTs are usually used in the blood for the cancer therapy, drug deliveries, and other important applications to the medication. The Medepalli et al.^9^, Benos et al.^10^, Yang et al.^11^, Alsagri et al.^12^ have used the blood based nanofluids for the various applications of medications.

Couple-stress fluids have wide range of applications in the industries as colloidal solutions, cooling processes, polymer fluids extraction etc.^13^. Contributions on the topic of nanofluid flow under different conditions are depicted in the articles^14–18^.

The study of electrically conducted incompressible fluid flows is termed as magnetohydrodynamic (MHD). The forces such as fluid’s Lorentz force have significant observations like the global magnetic field effects upon the Earth. This type of arena is certainly created with the help of strong Lorentz forces that are mainly present in Earth liquid core. Since the Lorentz forces are less common in our routine life observations that make the concept of magnetohydrodynamics difficult to understand. In present study, the role of Lorentz forces on fluids has established by taking electrically conducted fluid and propulsion of a magnetohydrodynamic ship. In review of^19^, this propulsion technique^20^ is attractive in several characteristics, as magnetohydrodynamic (MHD) propulsion does not need any movable parts. There are numerous applications of MHD propulsion as far as the high speed ships are concerned for naval submarines^21^. Baumgartl et al.^22^ have inspected the evaluation of MHD effects upon time dependent and independent fluid flow. In this work an exterior weak magnetic field has applied to the flow system. A detail analysis^23,24^ has carried out with main focus upon the impact of MHD fluid flow using different flow conditions and geometrical view of the flow system. The magnetohydrodynamic propulsion could be created in several techniques. As electro hydrodynamics is the study of electrically conducting ionized particles motions or atoms and their transportations with the neighboring liquids and electric fields. These particles, molecules or atom and liquid transportation process consists of electro-osmosis, electro-kinesis, electro-phoresis and electro rotation fusing metals^25^ in nuclear reactor and electric heater. Andersson^26^ has originated closed form solution for the incompressible fluid flow over the surface which was stretching. Over an exponential surface, the numerical and analytical solution for the incompressible fluid flow with the exponential jump of temperature has explained by Magyari and keller^27^. Partha et al.^28^ have studied the mutual influence of dissipation and convective flow past a stretched surface. The energy transportation and numerical simulation of viscous fluid flow past a stretching sheet has designed by Elbashbeshy^29^. Ellahi et al.^30^ have applied HAM method for the investigation of 3D flow of Carreau liquid using magnetic effects. Rashidi et al.^31^ have investigated the Burger’s model for nanofluid flow under the impact of magnetic effects.

In 1856 the Henry Darcy has investigated the flow of homogeneous fluids through madium, consisting of void spaces or pores (termed as porous medium). The work has carried out for small velosities and low permeable media. Later, Forchheimer^32^ has overcome the drowbackes of Darcy work by inserting the square of flow term in flow eqiuation. Muskat^33^ has identified the addtional term as ‘Forchheimer’ for the first time. Afterwards, a number of investigation have been conducted by differnt people using differnt geometries for fluid flow and heat transfer through porus media. Pal and Mondal^34^ have discussed the the mixed convection flow past a permeable medium with differnt flow conditions. Ganesh et al.^35^ have inspected the nanofluid flow past a shrinkng and stretching porous surface with application of second order slip condition. Seddeek^36^ has discussed the combined effects of themophoresis and viscous dissipation for investigation of mixed convective Darcy Forchheimer flow through a permeable surface. It has observed in this study that, the flow has reduced with augmentation in inertia cofficient and porosity parameter. Hayat et al.^37^ have used the Cattaneo Christove thermal flux model to Darcy Forchheimer flow with varying heat conductivity through porous surface. A number of viscoelastic models have been developed to explain the properties and behavior of non-Newtonian fluids, including the couple stress fluid model. These fluids' constitutive equations are frequently complicated, including a large number of parameters. The couple stress fluid model is the most simple modification of the classical theory of fluids, allowing for polar effects in the fluid medium such as couple stresses and body couples. A couple stress fluids are used in the chemical and engineering sectors. Sinha and Singh^38^ investigated the effects of couple stresses on blood flow via a narrow artery with slight stenosis.

In daily life, most of the physical phenomen are non-linear rather some are highly nonlinear phenomenon. Solution of such complex and complicated physical problems is very difficult, even in some cases it becomes impposible to obtaine the analytical solution. In order to solve such problems most of the investigators are emloying different numerical or analytical techniques. Out of such techniques, HAM^39–44^ is also useful for solution of such problems.

Principal aim of this research is to inspect the heat transmission and the influence of electro-magnetic effects upon MHD flow of a couple stress hybrid nanofluids over a Darcy-Forchheimer model in a symmetric flow with variable viscosity. The equations investigating the electro-magneto hydrodynamic of MHD flow for a hybrid fluid have converted to non-dimensional notation with suitable variables. The semi-analytical technique HAM is employed to solve the obtained set of equations. The impact of permeability, couple-stress and magnetic parameters on axial velocity, mean critical reflux condition and mean velocity on the channel walls are examined in details. The augmentation in the temperature distribution assesses the pH values and electric conductivity. Consequently, the $SWCNTs\,\,{\text{and}}\,\,MWCNTs$ hybrid nanofluids are utilized in this study for medication purpose.

## Mathematical modeling

**Figure 1:**
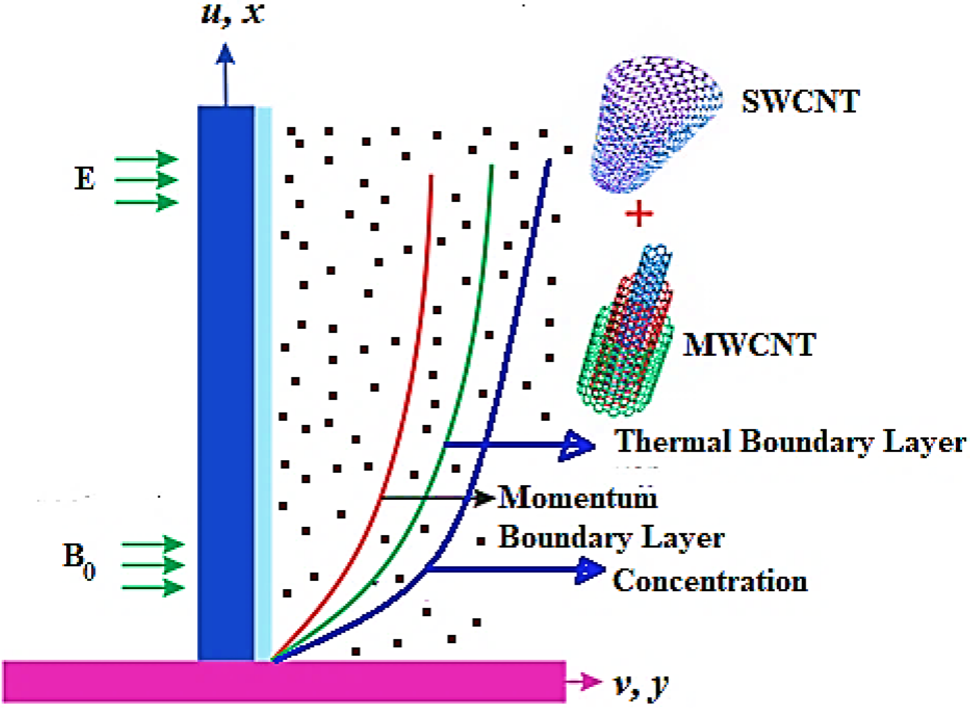
Geometry of the problem.

Take a two-dimensional electro-hydrodynamic flow of viscous liquid couple stress nanofluid past a stretching surface. The fluid is stabilized by the collective effects of electric and magnetic fields. The effects of Joule heating and viscous dissipation have also been considered for the current flow system in order to control its thermal characteristics. The mathematical expression for Lorentz force is described as $\vec{J} \times \vec{B}$ in which the magnetic field is represented by $\vec{B}$ while current density by $\vec{J}$. Mathematically the expression for $\vec{J}$ by Ohm’s law is described as $\vec{J}\,\,\, = \,\,\sigma \,\,\left( {\vec{E}\,\, + \,\,\vec{V}\,\, \times \,\,\vec{B}} \right)$. Where ‘$E$’ stands for electric field such that $\vec{E} = 0$, while the field electrical conductivity is given by ‘$\sigma$’. The temperature of nanofluid at surface of wall is $T_{w}$, whereas at the free stream it is $T_{\infty }$. The complete geometry of the current model is shown in Fig. 1.

By applying the above suppositions, resultant equations are:

1 $$ \frac{\partial v}{{\partial y}} + \frac{\partial u}{{\partial x}} = 0, $$

2 $$ \rho_{hnf} \left( {u\frac{\partial u}{{\partial x}} + v\frac{\partial u}{{\partial y}}} \right) = \frac{\partial }{\partial y}\left( {\mu_{hnf} (T)\frac{\partial u}{{\partial y}}} \right) + \sigma_{hnf} \left( {E_{0} B_{0} - B_{0}^{2} u} \right) - \frac{{\mu_{hnf} (T)}}{K}u - F_{0} u^{2} - \eta_{0} \frac{{\partial^{4} u}}{{\partial y^{4} }}, $$

3 $$ (\rho c_{p} )_{hnf} \left( {u\frac{\partial T}{{\partial x}} + v\frac{\partial T}{{\partial y}}} \right) = k_{hnf} \frac{{\partial T^{2} }}{{\partial y^{2} }} + \mu_{hnf} (T)\left( {\frac{\partial u}{{\partial y}}} \right)^{2} + \sigma_{hnf} \left( {uB_{0} - E_{0} } \right)^{2} + Q_{0} (T - T_{\infty } ), $$

4 $$ \left( {u\frac{\partial C}{{\partial x}} + v\frac{\partial C}{{\partial y}}} \right) = D_{m} \frac{{\partial C^{2} }}{{\partial y^{2} }} - k_{r} (C - C_{\infty } ), $$

The subjected conditions at boundary are:

5 $$ \begin{gathered} u = \,\,\,u_{w} \left( x \right)\,\,\, = \,\,\,bx,\,\,\,\,v\,\, = 0,\,\,\,T = T_{w} ,\,\,\,C = C_{w} \,\,\,\,at\,\,\,y = 0, \hfill \\ \,u = 0,\,\,\,v = 0,\,\,\,T \to T_{\infty } ,\,\,\,C \to C_{\infty } \,\,\,\,\,\,\,\,\,\,\,\,\,\,\,\,\,\,\,\,\,\,\,\,\,at\,\,y \to \infty . \hfill \\ \end{gathered} $$

Above $u, \, v$ are flow components along the x and y coordinate axes, $\rho_{hnf}$ is hybrid nanofluid density, $E_{0}$ is the strength of electric field, $B_{0}$ strength of magnetic field and $F_{0} = \frac{{C_{b} }}{\sqrt K }$ is Non-uniform inertia coefficient of porous medium. The kinematic viscosity is $\nu_{hnf}$, heat diffusivity is $\alpha_{hnf}$, heat capacity is $\left( {\rho c_{p} } \right)_{hnf}$, dynamic viscosity is given by $\mu_{hnf}$, and $Q_{0}$ is heat source. $C$ denotes nanoparticle concentration, $k_{r}$ chemical reaction.

### Thermophysical properties

The mathematical expression for thermophysical properties of base and hybrid nanofluid are described as^4–8^:

$$ \frac{{\mu (T)_{hnf} }}{{\mu_{f} }} = \frac{1}{{(1 - \phi_{1} )^{2.5} (1 - \phi_{2} )^{2.5} }}e^{{ - m(T - T_{\infty } )}} = \frac{{\mu_{hnf} }}{{\mu_{f} }}e^{{ - m(T - T_{\infty } )}} = \frac{{\mu_{hnf} }}{{\mu_{f} }}e^{\Lambda \Theta } , $$

7 $$ \frac{{\rho_{hnf} }}{{\rho_{f} }} = \left[ {\left( {1 - \phi_{2} } \right)\left\{ {1 - \left( {1 - \frac{{\rho_{MWCNTs} }}{{\rho_{f} }}} \right)\phi_{1} } \right\} + \phi_{2} \frac{{\rho_{SWCNTs} }}{{\rho_{f} }}} \right] $$

8 $$ \begin{gathered} \frac{{k_{hnf} }}{{k_{bf} }} = \left( {1 - \phi_{2} } \right) + 2\phi_{2} \left( {\frac{{k_{SWCNT} }}{{k_{SWCNT} - k_{bf} }}} \right)ln\left( {\frac{{k_{SWCNT} + k_{bf} }}{{2k_{bf} }}} \right), \hfill \\ \frac{{k_{bf} }}{{k_{f} }} = \left( {1 - \phi_{1} } \right) + 2\phi_{1} \left( {\frac{{k_{MWCNT} }}{{k_{MWCNT} - k_{f} }}} \right)ln\left( {\frac{{k_{MWCNT} + k_{f} }}{{2k_{f} }}} \right). \hfill \\ \end{gathered} $$

9 $$ \frac{{(\rho Cp)_{hnf} }}{{\left( {\rho Cp} \right)_{f} }} = \left[ {\left( {1 - \phi_{2} } \right)\left\{ {1 - \left( {1 - \frac{{\left( {\rho Cp} \right)_{MWCNTs} }}{{\left( {\rho Cp} \right)_{f} }}} \right)\phi_{1} } \right\} + \phi_{2} \frac{{\left( {\rho Cp} \right)_{SWCNTs} }}{{\left( {\rho Cp} \right)_{f} }}} \right] $$

The following appropriate variables are suggested:

10 $$ u = b\,\,x\,\,F^{\prime}\left( \eta \right),\,\,\,\,v = - \sqrt {b\,\nu } \,\,F\left( \eta \right),\,\,\,\Theta \left( \eta \right) = \frac{{T - T_{\infty } }}{{T_{w} - T_{\infty } }},\,\,\,\,\Phi \left( \eta \right) = \,\,\frac{{C - C_{\infty } }}{{C_{w} - C_{\infty } }},\,\,\,\eta = y\,\,\sqrt {\frac{b}{\nu }} , $$

Using Eq. (10) in Eqs. (1–5) we have

11 $$ F^{\prime\prime\prime} + \frac{{\rho_{hnf} }}{{\rho_{f} }}\frac{{\mu_{f} }}{{\mu_{hnf} }}e^{\Lambda \Theta } \left[ {FF^{\prime\prime} - (1 + Fr)F^{{\prime}{2}} } \right] + \frac{{\mu_{f} }}{{\mu_{hnf} }}e^{\Lambda \Theta } \left[ {M\left( {E - F^{\prime}} \right)^{2} - kF^{v} } \right] - \lambda F^{\prime} = 0, $$

12 $$ \frac{{k_{hnf} }}{{k_{f} }}\Theta^{\prime\prime} + \Pr \frac{{(\rho Cp)_{hnf} }}{{\left( {\rho Cp} \right)_{f} }}F\Theta^{\prime} + \frac{{\mu_{hnf} }}{{\mu_{f} }}e^{\Lambda \Theta } Ec\Pr \left( {F^{{\prime\prime}{2}} + M\left( {E - F^{\prime}} \right)^{2} } \right) + Q\Pr \Theta = 0, $$

13 $$ \Phi^{\prime\prime} + ScF\Phi^{\prime} - \gamma Sc\Phi = 0. $$

With inter-related boundary conditions:

14 $$ \begin{gathered} F\left( 0 \right) = 0,F^{\prime}\left( 0 \right) = 1,\Theta \left( 0 \right) = 1,\Phi \left( 0 \right) = 1 \hfill \\ \hfill \\ \,\,\,\,\,\,\,\,\,F\left( \infty \right) = 0,\Theta \left( \infty \right) = 0,\Phi \left( \infty \right) = 0. \hfill \\ \end{gathered} $$

In above equations the dimensionless couple stress parameter is $k$, the Prandtl number is $Pr$, $M$ is magnetic parameter,$Q$ is heat flux parameter,$E$ is electric field parameter and $Ec$ is Eckert number. The mathematical descriptions for these parameters are given as follows:

15 $$ E = \frac{{E_{0} }}{{B_{0} u_{w} }},Fr = \frac{{xC_{b} }}{{K^{{{\raise0.7ex\hbox{$1$} \!\mathord{\left/ {\vphantom {1 2}}\right.\kern-\nulldelimiterspace} \!\lower0.7ex\hbox{$2$}}}} }},M = \frac{{\sigma B_{0}^{2} }}{{b\rho_{f} }},Ec = \frac{{u_{w}^{2} }}{{c_{p} \left( {T_{w} - T_{\infty } } \right)}},\Pr = \frac{{\nu_{f} }}{{\alpha_{f} }},Q = \frac{{Q_{0} }}{{b\left( {\rho c_{p} } \right)_{f} }}. $$

### Engineering quantities

The Skin friction, heat and mass fluxes have numerous uses in engineering field. The mathematical expression for these quantities is given as:

16 $$ C_{fx} = \frac{{\tau_{w} }}{{\frac{1}{2}\rho_{hnf} \left( {u_{w} } \right)^{2} }},\,\,\,\,\,\,\,Nu_{x} = \frac{{xq_{w} }}{{k_{hnf} \left( {T_{w} - T_{\infty } } \right)}},\,\,\,\,\,Sh_{x} = \frac{{xj_{w} }}{{D_{hnf} \left( {T_{w} - T_{\infty } } \right)}}. $$

Incorporating Eq. (10) in Eq. (16) we have

17 $$ C_{fx} Re_{x}^{0.5} = \frac{2}{{\left( {1 - \phi_{1} } \right)^{2.5} \left( {1 - \phi_{2} } \right)^{2.5} }}F^{\prime\prime}\left( 0 \right),\,\,\,\,Nu_{x} Re_{x}^{ - 0.5} = - \frac{{k_{hnf} }}{{k_{f} }}\Theta^{\prime}\left( 0 \right),Sh_{x} Re_{x}^{ - 0.5} = - \Phi^{\prime}\left( 0 \right). $$

### Method of solution

To solve Eqs. (11–13) with the help of Eq. (14), the semi-analytical technique HAM has used. This method requires some starting values which are given mathematically as follows:

18 $$ \overset{\lower0.5em\hbox{$\smash{\scriptscriptstyle\frown}$}}{F} (\eta ) = 1 - e^{ - \eta } ,\overset{\lower0.5em\hbox{$\smash{\scriptscriptstyle\frown}$}}{\Theta } (\eta ){ = }e^{ - \eta } ,\overset{\lower0.5em\hbox{$\smash{\scriptscriptstyle\frown}$}}{\Phi } (\eta ){ = }e^{ - \eta } , $$

19 $$ L_{{\overset{\lower0.5em\hbox{$\smash{\scriptscriptstyle\frown}$}}{F} }} (\overset{\lower0.5em\hbox{$\smash{\scriptscriptstyle\frown}$}}{F} ) = \overset{\lower0.5em\hbox{$\smash{\scriptscriptstyle\frown}$}}{f^{\prime\prime\prime}} ,\,\,\,\,\,{\text{L}}_{{\overset{\lower0.5em\hbox{$\smash{\scriptscriptstyle\frown}$}}{\Theta } }} {(}\overset{\lower0.5em\hbox{$\smash{\scriptscriptstyle\frown}$}}{\Theta } {) = }\overset{\lower0.5em\hbox{$\smash{\scriptscriptstyle\frown}$}}{\Theta }^{\prime\prime},\,\,\,\,\,\,{\text{L}}_{{\overset{\lower0.5em\hbox{$\smash{\scriptscriptstyle\frown}$}}{\Phi } }} {(}\overset{\lower0.5em\hbox{$\smash{\scriptscriptstyle\frown}$}}{\Phi } {) = }\overset{\lower0.5em\hbox{$\smash{\scriptscriptstyle\frown}$}}{\Phi }^{\prime\prime}\,\,, $$

The operators $$L_{{\overset{\lower0.5em\hbox{$\smash{\scriptscriptstyle\frown}$}}{F} }} ,\,\,{\text{L}}_{{\overset{\lower0.5em\hbox{$\smash{\scriptscriptstyle\frown}$}}{\Theta } }}$$, $${\text{L}}_{{\overset{\lower0.5em\hbox{$\smash{\scriptscriptstyle\frown}$}}{\Phi } }}$$ are given as

20 $$ L_{{\overset{\lower0.5em\hbox{$\smash{\scriptscriptstyle\frown}$}}{F} }} (e_{1} + e_{2} \eta + e_{3} \eta^{2} )\,\,\, = \,\,\,0,\,\,\,\,\,{\text{L}}_{{\overset{\lower0.5em\hbox{$\smash{\scriptscriptstyle\frown}$}}{\Theta } }} (e_{4} + e_{5} \eta )\,\,\, = \,\,0,\,\,\,\,\,\,\,\,{\text{L}}_{{\overset{\lower0.5em\hbox{$\smash{\scriptscriptstyle\frown}$}}{\Phi } }} (e_{6} + e_{7} \eta )\,\, = \,\,0. $$

The nonlinear operators $${\rm N}_{{\overset{\lower0.5em\hbox{$\smash{\scriptscriptstyle\frown}$}}{F} }} \,{,}\,\,{\rm N}_{{\overset{\lower0.5em\hbox{$\smash{\scriptscriptstyle\frown}$}}{\Theta } }} {\text{ and}}\,{\rm N}_{{\overset{\lower0.5em\hbox{$\smash{\scriptscriptstyle\frown}$}}{\Phi } }}$$ are expressed as:

21 $$ \begin{aligned}  {\rm N}_{{\overset{\lower0.5em\hbox{$\smash{\scriptscriptstyle\frown}$}}{F} }} \, \left[ {\overset{\lower0.5em\hbox{$\smash{\scriptscriptstyle\frown}$}}{F} (\eta ;\zeta )} \right] &  =  \overset{\lower0.5em\hbox{$\smash{\scriptscriptstyle\frown}$}}{F}_{\eta \eta \eta } { + }\frac{{\rho_{hnf} }}{{\rho_{f} }}\frac{{\mu_{f} }}{{\mu_{hnf} }}e^{\Lambda \Theta } \left[ {\overset{\lower0.5em\hbox{$\smash{\scriptscriptstyle\frown}$}}{F} \overset{\lower0.5em\hbox{$\smash{\scriptscriptstyle\frown}$}}{F}_{\eta \eta } - (1 + Fr)\overset{\lower0.5em\hbox{$\smash{\scriptscriptstyle\frown}$}}{F}_{\eta \eta }^{2} } \right]\\&\quad + \frac{{\mu_{f} }}{{\mu_{hnf} }}e^{\Lambda \Theta } \left[ {M\left( {E - \overset{\lower0.5em\hbox{$\smash{\scriptscriptstyle\frown}$}}{F}_{\eta } } \right)^{2} - k\overset{\lower0.5em\hbox{$\smash{\scriptscriptstyle\frown}$}}{F}_{\eta \eta \eta \eta \eta } } \right] - \lambda \overset{\lower0.5em\hbox{$\smash{\scriptscriptstyle\frown}$}}{F}_{\eta } , \hfill \\ \end{aligned} $$

22 $$ \begin{aligned} {\rm N}_{{\overset{\lower0.5em\hbox{$\smash{\scriptscriptstyle\frown}$}}{\Theta } }} \left[ {\overset{\lower0.5em\hbox{$\smash{\scriptscriptstyle\frown}$}}{F} (\eta ;\zeta ),\overset{\lower0.5em\hbox{$\smash{\scriptscriptstyle\frown}$}}{\Theta } (\eta ;\zeta )} \right] & = \frac{{k_{hnf} }}{{k_{f} }}\overset{\lower0.5em\hbox{$\smash{\scriptscriptstyle\frown}$}}{\Theta }_{\eta \eta } { + }\Pr \frac{{(\rho Cp)_{hnf} }}{{\left( {\rho Cp} \right)_{f} }}\overset{\lower0.5em\hbox{$\smash{\scriptscriptstyle\frown}$}}{F} \overset{\lower0.5em\hbox{$\smash{\scriptscriptstyle\frown}$}}{\Theta }_{\eta }\\&\quad + \frac{{\mu_{hnf} }}{{\mu_{f} }}e^{\Lambda \Theta } Ec\Pr \left( {\overset{\lower0.5em\hbox{$\smash{\scriptscriptstyle\frown}$}}{F}_{{^{\eta \eta } }}^{2} + M\left( {E - \overset{\lower0.5em\hbox{$\smash{\scriptscriptstyle\frown}$}}{F}_{\eta } } \right)^{2} } \right) + Q\Pr \overset{\lower0.5em\hbox{$\smash{\scriptscriptstyle\frown}$}}{\Theta } ,  \end{aligned} $$

23 $$ {\rm N}_{{\overset{\lower0.5em\hbox{$\smash{\scriptscriptstyle\frown}$}}{\Phi } }} \left[ {\overset{\lower0.5em\hbox{$\smash{\scriptscriptstyle\frown}$}}{F} (\eta ;\zeta ),\overset{\lower0.5em\hbox{$\smash{\scriptscriptstyle\frown}$}}{\Phi } (\eta ;\zeta )} \right] = \left( {1 - \phi_{1} } \right)\left( {1 - \phi_{2} } \right)\overset{\lower0.5em\hbox{$\smash{\scriptscriptstyle\frown}$}}{\Phi }_{\eta \eta } { + }Sc\overset{\lower0.5em\hbox{$\smash{\scriptscriptstyle\frown}$}}{F} \overset{\lower0.5em\hbox{$\smash{\scriptscriptstyle\frown}$}}{\Phi }_{\eta } - \gamma Sc\overset{\lower0.5em\hbox{$\smash{\scriptscriptstyle\frown}$}}{\Phi } , $$

For Eqs. (6 and 7) the 0th-order equations are

$$ (1 - \zeta )\,\,L_{{\overset{\lower0.5em\hbox{$\smash{\scriptscriptstyle\frown}$}}{F} }} \left[ {\overset{\lower0.5em\hbox{$\smash{\scriptscriptstyle\frown}$}}{F} (\eta ;\,\,\,\zeta )\,\,\, - \,\,\,\,\overset{\lower0.5em\hbox{$\smash{\scriptscriptstyle\frown}$}}{F}_{0} (\eta )} \right] = p\,\,\,\hbar_{{\overset{\lower0.5em\hbox{$\smash{\scriptscriptstyle\frown}$}}{F} }} \,\,{\rm N}_{{\overset{\lower0.5em\hbox{$\smash{\scriptscriptstyle\frown}$}}{F} }} \,\,\left[ {\overset{\lower0.5em\hbox{$\smash{\scriptscriptstyle\frown}$}}{F} (\eta ;\,\,\,\zeta )} \right] $$

25 $$ (1 - \zeta ) \, L_{{\overset{\lower0.5em\hbox{$\smash{\scriptscriptstyle\frown}$}}{\Theta } }} \left[ {\overset{\lower0.5em\hbox{$\smash{\scriptscriptstyle\frown}$}}{\Theta } \,\,(\eta \,\,;\,\,\,\zeta ) - \overset{\lower0.5em\hbox{$\smash{\scriptscriptstyle\frown}$}}{\Theta }_{0} \,\,(\eta )} \right]\,\,\, = \,\,p\hbar_{{\overset{\lower0.5em\hbox{$\smash{\scriptscriptstyle\frown}$}}{\Theta } }} {\rm N}_{{\overset{\lower0.5em\hbox{$\smash{\scriptscriptstyle\frown}$}}{\Theta } }} \left[ {F(\eta \,\,;\,\,\zeta ),\overset{\lower0.5em\hbox{$\smash{\scriptscriptstyle\frown}$}}{\Theta } (\eta \,\,;\,\,\zeta )} \right] $$

26 $$ (1 - \zeta ) \, L_{{\overset{\lower0.5em\hbox{$\smash{\scriptscriptstyle\frown}$}}{\Phi } }} \left[ {\overset{\lower0.5em\hbox{$\smash{\scriptscriptstyle\frown}$}}{\Phi } (\eta \,\,;\,\,\zeta )\,\,\, - \,\,\,\overset{\lower0.5em\hbox{$\smash{\scriptscriptstyle\frown}$}}{\Phi }_{0} (\eta )} \right] = p\,\,\hbar_{{\overset{\lower0.5em\hbox{$\smash{\scriptscriptstyle\frown}$}}{\Phi } }} {\rm N}_{{\overset{\lower0.5em\hbox{$\smash{\scriptscriptstyle\frown}$}}{\Phi } }} \left[ {F(\eta \,\,\,;\,\,\zeta ),\overset{\lower0.5em\hbox{$\smash{\scriptscriptstyle\frown}$}}{\Phi } \,\,(\eta \,\,;\,\,\zeta )} \right] $$

While BCs are

27 $$ \begin{gathered} \left. {\overset{\lower0.5em\hbox{$\smash{\scriptscriptstyle\frown}$}}{F} (\eta \,\,\,;\,\,\,\zeta )} \right|_{\eta = 0} = 0, \, \,\,\,\,\,\,\,\,\,\,\,\,\,\,\left. {\frac{{\partial \overset{\lower0.5em\hbox{$\smash{\scriptscriptstyle\frown}$}}{F} (\eta \,\,;\,\,\zeta )}}{\partial \eta }} \right|_{\eta = 0} = 1, \, \hfill \\ \left. {\overset{\lower0.5em\hbox{$\smash{\scriptscriptstyle\frown}$}}{\Theta } (\eta \,\,\,;\,\,\zeta )} \right|_{\eta = 0} = 1,\left. {\,\,\,\,\,\,\,\,\,\,\,\,\,\,\,\,\,\,\,\,\,\overset{\lower0.5em\hbox{$\smash{\scriptscriptstyle\frown}$}}{\Phi } (\eta \,\,;\,\,\zeta )} \right|_{\eta = 0} = 1, \, \hfill \\ \, \left. {\overset{\lower0.5em\hbox{$\smash{\scriptscriptstyle\frown}$}}{F} (\eta \,\,;\,\,\zeta )} \right|_{\eta = \infty } \to 0,\left. {\,\,\,\,\,\,\,\,\,\,\,\overset{\lower0.5em\hbox{$\smash{\scriptscriptstyle\frown}$}}{\Theta } (\eta \,\,;\,\,\zeta )} \right|_{\eta = \infty } \to 0,\,\,\,\,\,\,\left. {\overset{\lower0.5em\hbox{$\smash{\scriptscriptstyle\frown}$}}{\Phi } (\eta \,\,;\,\,\zeta )} \right|_{\eta = \infty } \to 0, \hfill \\ \end{gathered} $$

Moreover, we further have

28 $$ \overset{\lower0.5em\hbox{$\smash{\scriptscriptstyle\frown}$}}{F} (\eta \,\,\,;\,\,1) = \overset{\lower0.5em\hbox{$\smash{\scriptscriptstyle\frown}$}}{F} (\eta ),\,\,\,\overset{\lower0.5em\hbox{$\smash{\scriptscriptstyle\frown}$}}{\Theta } (\eta \,\,;\,\,1) = \overset{\lower0.5em\hbox{$\smash{\scriptscriptstyle\frown}$}}{\Theta } (\eta ),\,\,\,\,\overset{\lower0.5em\hbox{$\smash{\scriptscriptstyle\frown}$}}{\Phi } (\eta \,\,;\,\,1) = \overset{\lower0.5em\hbox{$\smash{\scriptscriptstyle\frown}$}}{\Phi } (\eta ). $$

The Taylor’s expansion for $$\overset{\lower0.5em\hbox{$\smash{\scriptscriptstyle\frown}$}}{F} (\eta \,\,;\,\,\zeta ) \, $$ and $$\overset{\lower0.5em\hbox{$\smash{\scriptscriptstyle\frown}$}}{\Theta } (\eta \,\,;\,\,\zeta ),\,\,\overset{\lower0.5em\hbox{$\smash{\scriptscriptstyle\frown}$}}{\Phi } (\eta \,\,;\,\,\,\zeta )$$ about $$\zeta = 0$$ are given as follows

29 $$ \begin{gathered} \overset{\lower0.5em\hbox{$\smash{\scriptscriptstyle\frown}$}}{F} (\eta \,\,\,;\,\,\zeta ) \, = \, \overset{\lower0.5em\hbox{$\smash{\scriptscriptstyle\frown}$}}{F}_{0} (\eta ) + \sum\nolimits_{n = 1}^{\infty } {\,\,\,\overset{\lower0.5em\hbox{$\smash{\scriptscriptstyle\frown}$}}{F}_{n} (\eta )\,\,\,\zeta^{n} } , \hfill \\ \overset{\lower0.5em\hbox{$\smash{\scriptscriptstyle\frown}$}}{\Theta } (\eta \,\,\,;\,\,\,\zeta ) \, = \, \overset{\lower0.5em\hbox{$\smash{\scriptscriptstyle\frown}$}}{\Theta }_{0} (\eta ) + \sum\nolimits_{n = 1}^{\infty } {\,\,\,\overset{\lower0.5em\hbox{$\smash{\scriptscriptstyle\frown}$}}{\Theta }_{n} (\eta )\,\,\zeta^{n} } , \hfill \\ \overset{\lower0.5em\hbox{$\smash{\scriptscriptstyle\frown}$}}{\Phi } (\eta \,\,\,;\,\,\zeta ) \, = \, \overset{\lower0.5em\hbox{$\smash{\scriptscriptstyle\frown}$}}{\Phi }_{0} (\eta ) + \sum\nolimits_{n = 1}^{\infty } {\,\,\,\overset{\lower0.5em\hbox{$\smash{\scriptscriptstyle\frown}$}}{\Phi }_{n} (\eta )\,\,\,\zeta^{n} .} \hfill \\ \end{gathered} $$

30 $$ \overset{\lower0.5em\hbox{$\smash{\scriptscriptstyle\frown}$}}{F}_{n} (\eta ) \, = \left. {\frac{1}{n!}\frac{\partial (\eta ;\zeta )}{{\partial \eta }}} \right|_{p = 0} ,\overset{\lower0.5em\hbox{$\smash{\scriptscriptstyle\frown}$}}{\Theta }_{n} (\eta ) \, = \left. {\frac{1}{n!}\frac{{\partial \overset{\lower0.5em\hbox{$\smash{\scriptscriptstyle\frown}$}}{\Phi } (\eta ;\zeta )}}{\partial \eta }} \right|_{p = 0} ,\overset{\lower0.5em\hbox{$\smash{\scriptscriptstyle\frown}$}}{\Phi } (\eta ) = \overset{\lower0.5em\hbox{$\smash{\scriptscriptstyle\frown}$}}{\Phi } (\eta ) \, = \left. {\frac{1}{n!}\frac{{\partial \overset{\lower0.5em\hbox{$\smash{\scriptscriptstyle\frown}$}}{\Phi } (\eta ;\zeta )}}{\partial \eta }} \right|_{p = 0} . $$

While BCs are:

31 $$ \begin{gathered} \overset{\lower0.5em\hbox{$\smash{\scriptscriptstyle\frown}$}}{F} \left( 0 \right) = 0,\,\,\,\overset{\lower0.5em\hbox{$\smash{\scriptscriptstyle\frown}$}}{F^{\prime}} \left( 0 \right) = 1,\,\,\,\overset{\lower0.5em\hbox{$\smash{\scriptscriptstyle\frown}$}}{\Theta } \left( 0 \right) = 1,\,\,\,\,\overset{\lower0.5em\hbox{$\smash{\scriptscriptstyle\frown}$}}{\Phi } \left( 0 \right) = 1, \hfill \\ \hfill \\ \overset{\lower0.5em\hbox{$\smash{\scriptscriptstyle\frown}$}}{F^{\prime}} \left( \eta \right) \to 0,\,\,\,\,\overset{\lower0.5em\hbox{$\smash{\scriptscriptstyle\frown}$}}{\Theta } \left( \eta \right) \to 0,\,\,\,\,\overset{\lower0.5em\hbox{$\smash{\scriptscriptstyle\frown}$}}{\Phi } \left( \eta \right) \to 0,\,\,\,\,\,\,\,\eta \to \infty . \hfill \\ \end{gathered} $$

Now

32 $$ \begin{gathered} \Re_{n}^{{\overset{\lower0.5em\hbox{$\smash{\scriptscriptstyle\frown}$}}{F} }} \left( \eta \right) = \overset{\lower0.5em\hbox{$\smash{\scriptscriptstyle\frown}$}}{F^{\prime\prime\prime}}_{n - 1} \hfill \\ + \frac{{\rho_{hnf} }}{{\rho_{f} }}\frac{{\mu_{f} }}{{\mu_{hnf} }}e^{\Lambda \Theta } \left[ {\sum\limits_{j = 0}^{w - 1} {\overset{\lower0.5em\hbox{$\smash{\scriptscriptstyle\frown}$}}{F}_{w - 1 - j} \overset{\lower0.5em\hbox{$\smash{\scriptscriptstyle\frown}$}}{F^{\prime\prime}}_{j} } - (1 + Fr)\overset{\lower0.5em\hbox{$\smash{\scriptscriptstyle\frown}$}}{F^{\prime}}_{{^{n - 1} }}^{2} } \right] + \frac{{\mu_{f} }}{{\mu_{hnf} }}e^{\Lambda \Theta } \left[ {M\left( {E - F^{\prime}_{n - 1} } \right)^{2} - kF_{{^{n - 1} }}^{v} } \right] - \lambda F^{\prime}_{n - 1} = 0, \hfill \\ \end{gathered} $$

33 $$ \begin{gathered} \Re_{n}^{{\overset{\lower0.5em\hbox{$\smash{\scriptscriptstyle\frown}$}}{\Theta } }} (\eta ) = \frac{{k_{hnf} }}{{k_{f} }}\overset{\lower0.5em\hbox{$\smash{\scriptscriptstyle\frown}$}}{\Theta^{\prime\prime}}_{n - 1} + \Pr \frac{{(\rho Cp)_{hnf} }}{{\left( {\rho Cp} \right)_{f} }}\sum\limits_{j = 0}^{w - 1} {\overset{\lower0.5em\hbox{$\smash{\scriptscriptstyle\frown}$}}{F}_{w - 1 - j} } \overset{\lower0.5em\hbox{$\smash{\scriptscriptstyle\frown}$}}{\Theta^{\prime}}_{j} \hfill \\ + \frac{{\mu_{hnf} }}{{\mu_{f} }}e^{{\Lambda \overset{\lower0.5em\hbox{$\smash{\scriptscriptstyle\frown}$}}{\Theta } }} Ec\Pr \left( {\overset{\lower0.5em\hbox{$\smash{\scriptscriptstyle\frown}$}}{F^{\prime\prime}}_{{^{n - 1} }}^{2} + M\left( {E - \overset{\lower0.5em\hbox{$\smash{\scriptscriptstyle\frown}$}}{F^{\prime}}_{n - 1} } \right)^{2} } \right) + Q\Pr \overset{\lower0.5em\hbox{$\smash{\scriptscriptstyle\frown}$}}{\Theta }_{n - 1} = 0, \hfill \\ \end{gathered} $$

34 $$ \Re_{n}^{{\overset{\lower0.5em\hbox{$\smash{\scriptscriptstyle\frown}$}}{\Phi } }} (\eta ) = \left( {1 - \phi_{1} } \right)\left( {1 - \phi_{2} } \right)\overset{\lower0.5em\hbox{$\smash{\scriptscriptstyle\frown}$}}{\Phi }^{\prime\prime}_{n - 1} + Sc\sum\limits_{j = 0}^{w - 1} {\overset{\lower0.5em\hbox{$\smash{\scriptscriptstyle\frown}$}}{F}_{w - 1 - j} } \overset{\lower0.5em\hbox{$\smash{\scriptscriptstyle\frown}$}}{\Phi }^{\prime}_{j} - \gamma Sc\overset{\lower0.5em\hbox{$\smash{\scriptscriptstyle\frown}$}}{\Phi }_{n - 1} = 0. $$

While

35 $$ \chi_{n} = \left\{ \begin{gathered} 0,{\text{ if }}\zeta \le {1} \hfill \\ 1,{\text{ if }}\zeta > {1}{\text{.}} \hfill \\ \end{gathered} \right. $$

## Discussion of results

In this work we have thoroughly inspected the flow of fluid and transmission of heat for a couple stress nanoliquid inserted among the viscous fluid packed through a horizontal conduit. We shall discuss the impact of different physical factors upon the fluid flow system in following paragraphs with the help of graphical view.

### Velocity profile

**Figure 2:**
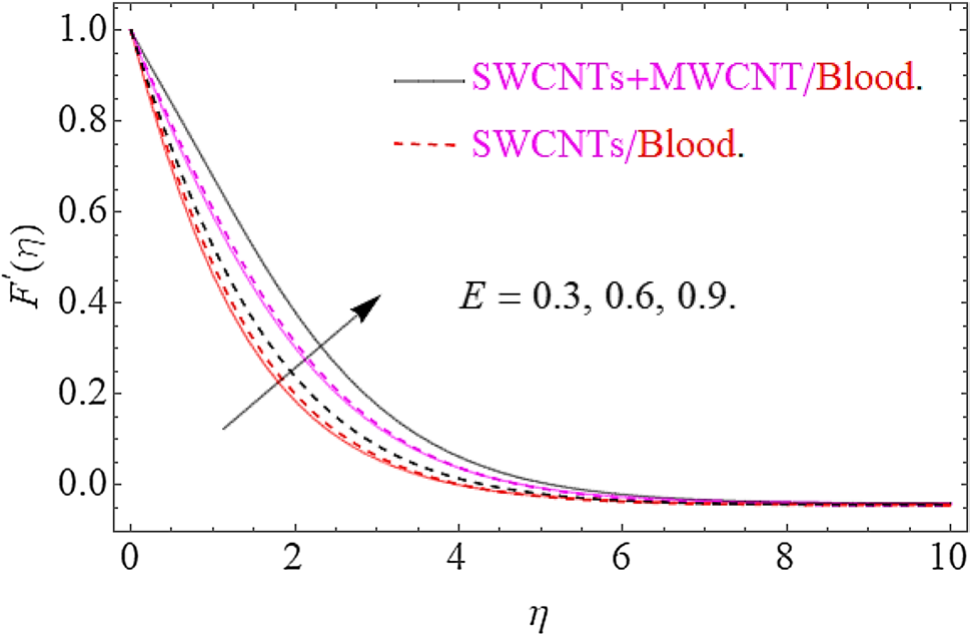
Effects of $E$ on $F^{\prime}\left( \eta \right)$.

**Figure 3:**
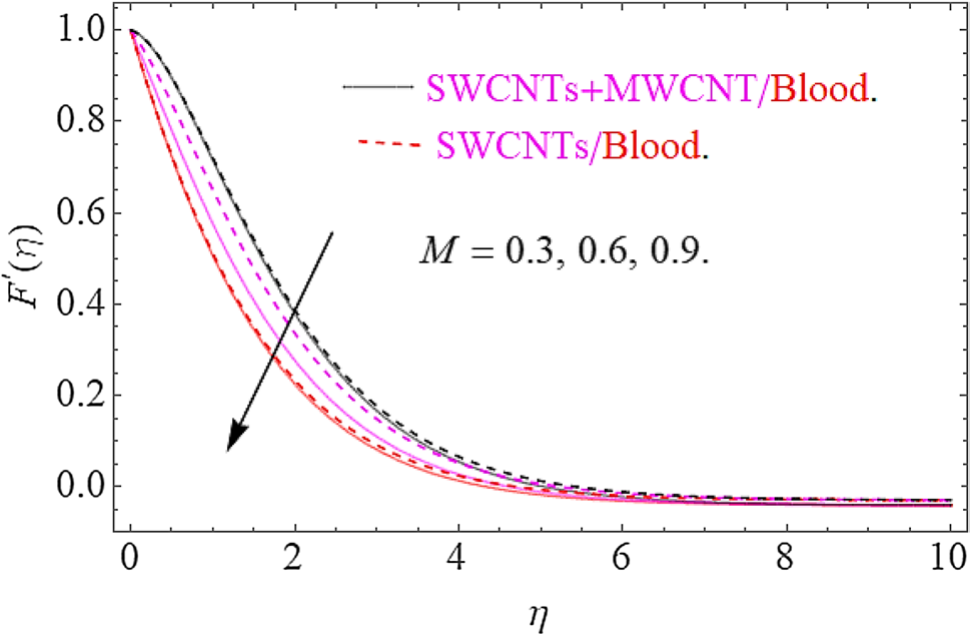
Impact of $M$ on $F^{\prime}\left( \eta \right)$.

**Figure 4:**
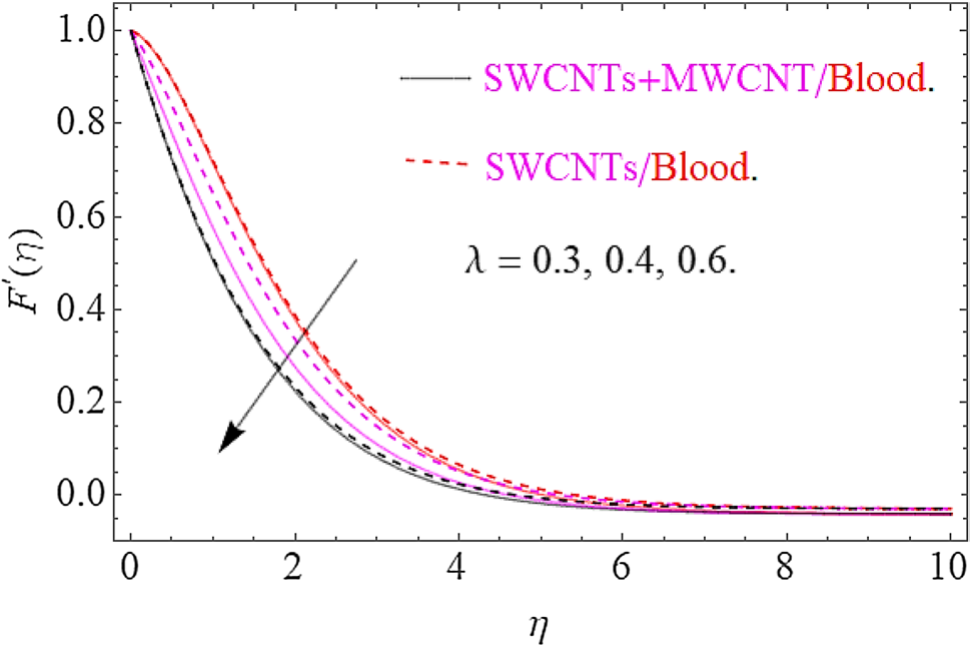
Impact of $\lambda$ on $F^{\prime}\left( \eta \right)$.

**Figure 5:**
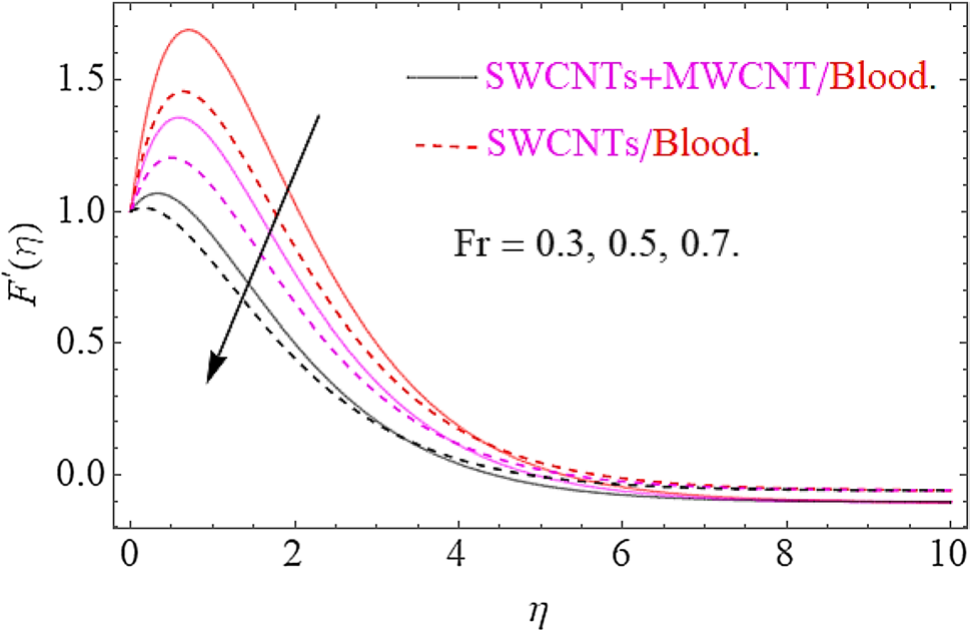
Influence of $Fr$ on $F^{\prime}\left( \eta \right)$.

**Figure 6:**
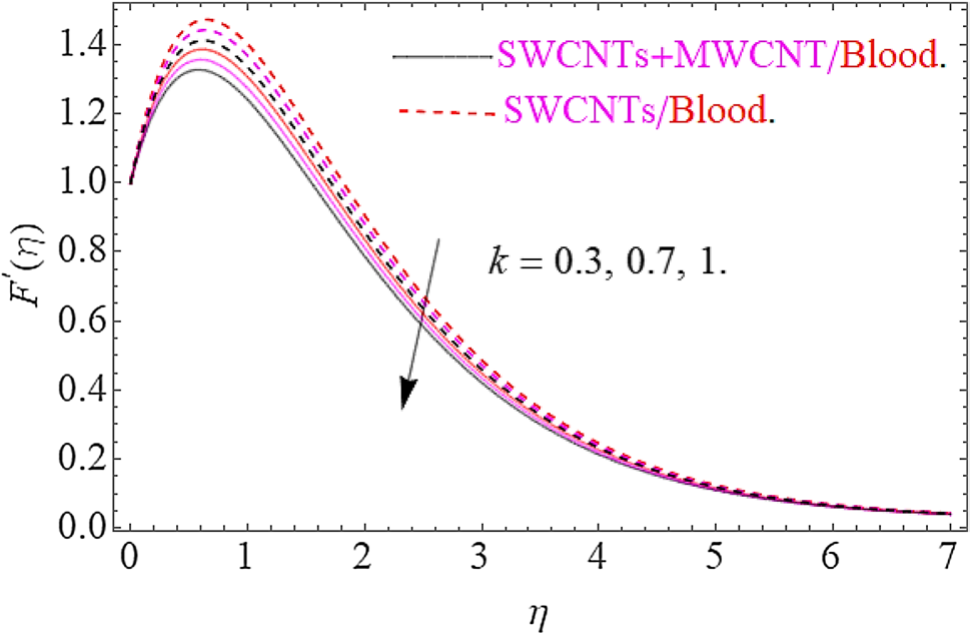
Effects of $k$ on $F^{\prime}\left( \eta \right)$.

The influence of different physical parameters such as $E,M,\lambda \,,F_{r} ,k$ upon flow field $F^{\prime}\left( \eta \right)$ has presented in Figs. 2, 3, 4, 5 and 6. Figure 2 exposed the effect of electric field on velocity field $F^{\prime}\left( \eta \right)$. In the presence of electrical effects, the field of velocity decreases and at a certain distance away from the wall, it rises closed to the nonlinear stretching sheet. In Fig. 3 we found that, the magnetic effect has reduced the flow field. Actually, for increasing values of $M$ there is a generation of Lorentz force in opposite direction of flow field that declines the velocity filed. This physical phenomenon augments the thermal and concentration characteristics. The effect of porosity parameter λ shows a decrement in the flow profile as depicted in Fig. 4. Physically, for increasing values of $\lambda$, the void spaces in the medium augments, that offers more resistive force to fluid motion. In this process the flow of fluid declines. Figure 5 depicts the impact upon flow profile for augmentation in inertia coefficient $F_{r}$. From this figure, it has perceived that an augmentation in $F_{r}$ results in generation of resistive force to fluid motion, that result a reduction in flow profile. Figure 6 calculates the influence of $k$ upon flow profile. It has revealed from this figure that, for augmenting values of $k$ the viscous forces will jump up that acts as a reducing agent to fluid motion. Hence the flow profile declines for growth in $k$.

### Thermal profile

**Figure 7:**
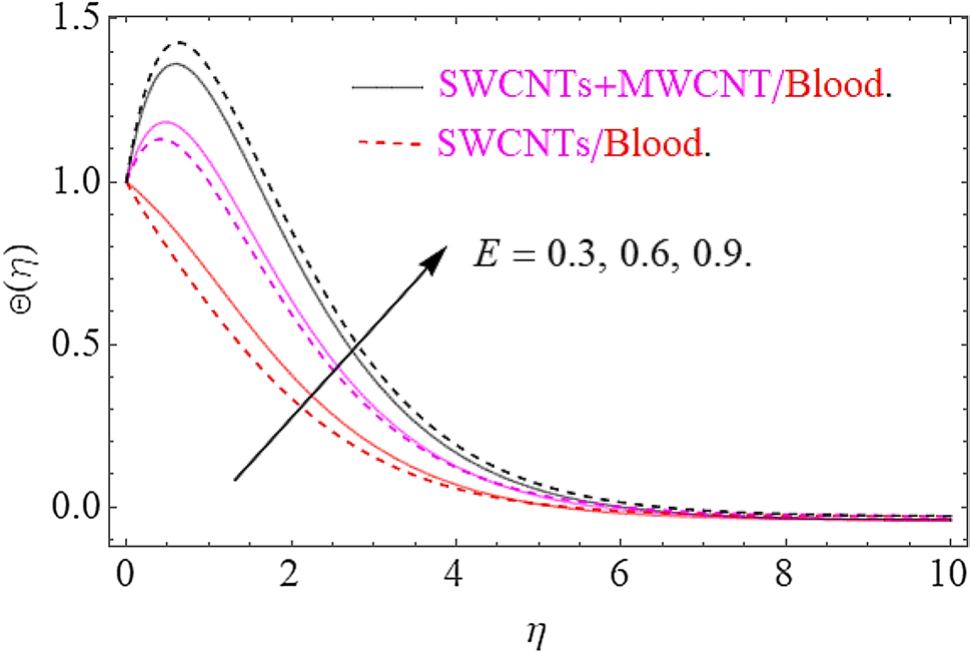
Impact of $E$ on $\Theta \left( \eta \right)$.

**Figure 8:**
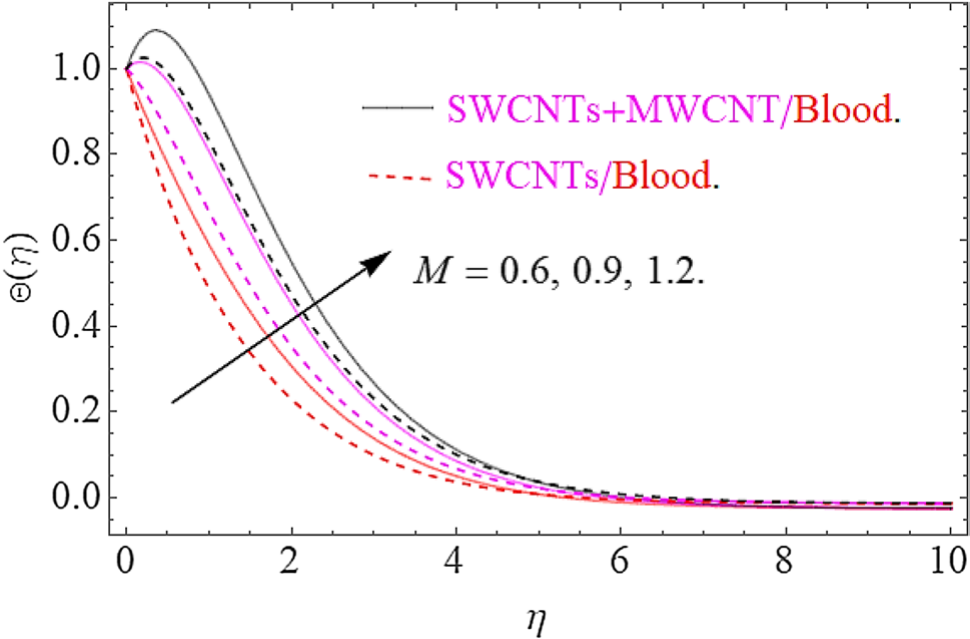
effects of $M$ on $\Theta \left( \eta \right)$.

**Figure 9:**
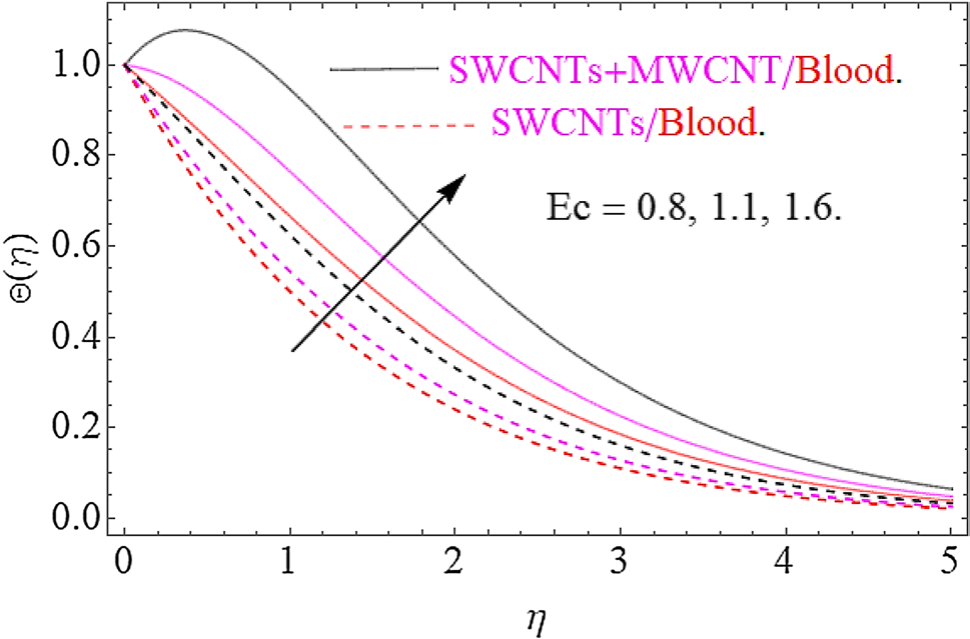
Impact of $Ec$ on $\Theta \left( \eta \right)$.

**Figure 10:**
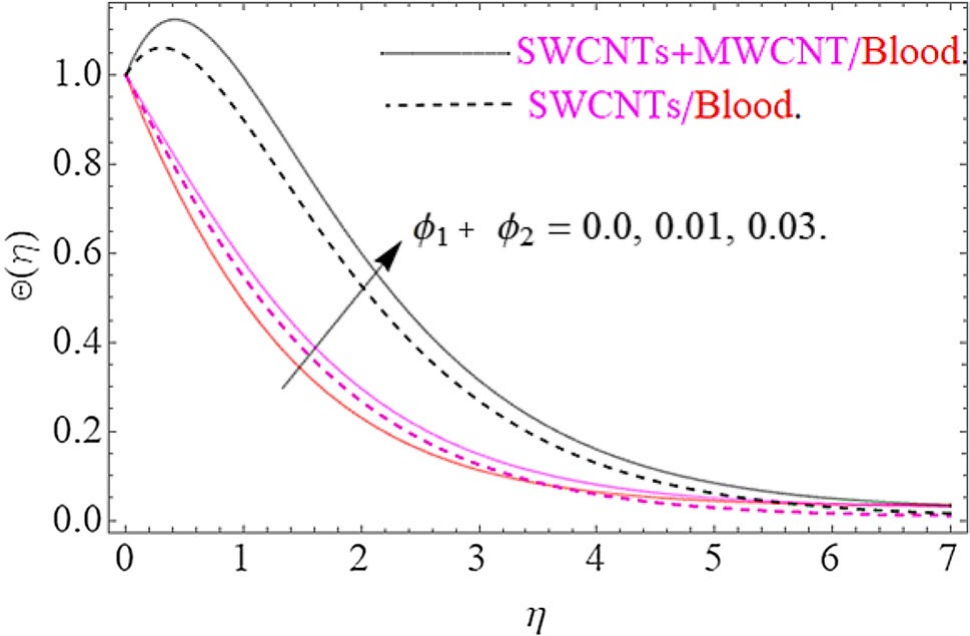
Effects of $\phi_{1} = \phi_{2}$ on $\Theta \left( \eta \right)$.

Impact of substantial parameters $\Pr ,E,M,Ec,\phi_{1} = \phi_{2}$ on temperature variation $\theta \left( \eta \right)$ is shown in Figs. 7, 8, 9 and 10. The effects of electric filed $E$ depict in Fig. 7. Clearly the electric filed is directly proportional to the rise in temperature. Hence increase in $E$ accelerates the Lorentz force due to which temperature of nanoparticles increases. Figure 8 depicts that hike in magnetic parameter corresponds to an augmentation in temperature profile. Physically, higher values of $M$ pushes the Lorentz force that creates a resistance to the fluid motion. In this phenomenon the thermal profile jumps up. The increase in Eckert number $Ec$ causes an augmentation in thermal flow as portrays in Fig. 9. Actually, due to higher values of $Ec$ the fluid friction amongst nanoparticles generates with more intensity. In this physical phenomenon the kinetic energy transformed to thermal energy that finally supports the augmentation in thermal profile. Similarly, augmenting values of volumetric friction causes increase in the dense behavior of the fluid particles. In this physical procedure the flow of fluid depreciates and thermal behavior of fluid particles appreciates. Hence the higher values of $\phi_{1} = \phi_{2}$ corresponds to augmentation in thermal profile as depicted in Fig. 10.

### Concentration profiles

**Figure 11:**
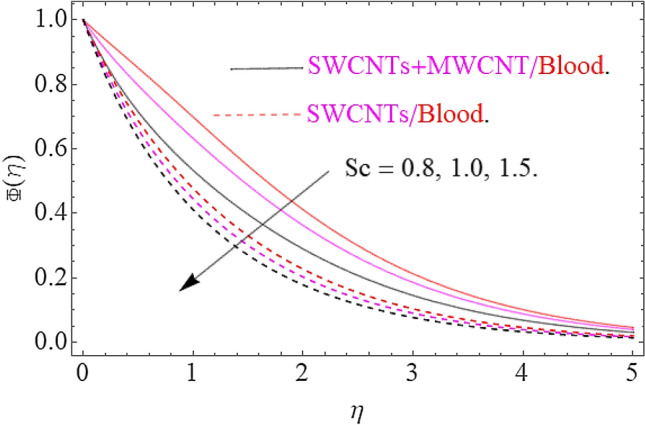
Impact of $Sc$ on $\Phi \left( \eta \right)$.

**Figure 12:**
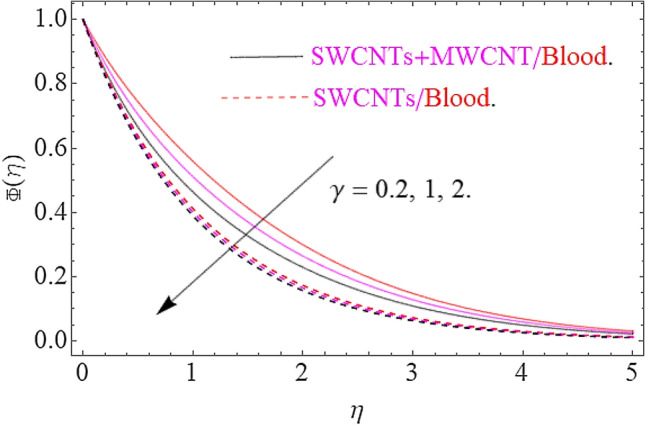
Influence of $\gamma$ on $\Phi \left( \eta \right)$.

**Figure 13:**
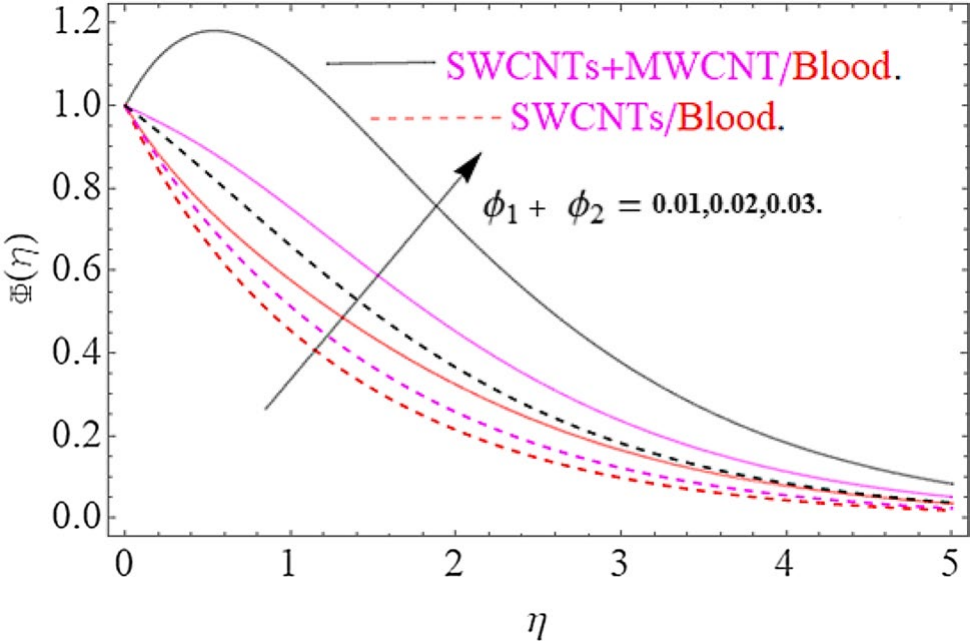
Impact of $\phi_{1} = \phi_{2}$ on $\Phi \left( \eta \right)$.

The result of physical parameters $Sc,\gamma ,\phi_{1} = \phi_{2}$ has been observed for concentration distribution $\phi \left( \eta \right)$ in Figs. 11, 12 and 13. Influence of Schmidt number upon dimensionless concentration profile is displayed through Fig. 11. This figure illustrates that an upsurge in Schmidt number with respect to a weaker solute diffusion allows a deep penetration of solutal effect. As a result, the concentration decreases with increase in Sc. Figure 12 depicts that, rising in the chemical reaction parameter rapidly reduces the concentration profile. The major reason is that, the number of molecules of solute involved a chemical reaction increase with rise in chemical reaction parameter, which results in decrease of concentration field. Moreover, it is verified that the concentration of profile becomes sharp for augmentation in chemical reaction. The Fig. 13 clearly shows the effects of volume friction upon concentration. Physically, the thermal conductivity increases by boosting the volume concentration of nanoparticles and as a result the nanoparticles act as bridge to pass the heat flow. In this physical phenomenon the concentration profile jumps up as depicted in Fig. 13.

### Tables discussion

The physical parameters influenced on the drag force for the nanofluid and hybrid nanofluid have been demonstrated in Table 1. The higher magnitude of parameters $\phi_{1} + \phi_{2} ,\Lambda ,M,k,Fr,\lambda$ increasing the skin friction coefficient and these consequences are relatively larger in the hybrid nanofluids. The electric field parameter declines the drag force for its increasing value and very small difference between the traditional and hybrid nanofluids has obtained. The rate of heat transfer for nanofluid and hybrid nanofluid versus different parameters has been displayed in Table 2. The augmentation in $\phi_{1} + \phi_{2} ,M,Q,Ec$ is enhancing the rate of thermal transmission while the increasing value for viscosity parameter $\Lambda$ declines this physical quantity. The characteristics of the Schmitt number $Sc$, chemical reaction parameter $\gamma$ and nanoparticle volume fraction $\phi_{1} ,\phi_{2}$ are displayed in Table 3. It has been revealed that greater values of these parameters have augmented the concentration rate.

**Table 1:** Skin friction $C_{fx} Re_{x}^{0.5}$ for different parameters regarding blood-based hybrid nanofluid.

$\Lambda$	$k$	$\phi_{1} + \phi_{2}$	$M$	$E$	$Fr$	$\lambda$	$SWCNTs - Cf_{x}$	$SWCNTs + MWCNTs - Cf_{x}$
0.2	0.1	0.01	0.2	0.2	0.2	0.2	1.01150	1.023411
0.4							1.12242	1.13451
0.5							1.23231	1.243442
	0.3						1.24024	1.264234
	0.5						1.26103	1.2832161
		0.02					1.22342	1.2343212
		0.03					1.32512	1.336242
			0.4				1.431272	1.442461
			0.6				1.4423625	1.4535219
				0.4			1.4364210	1.42432041
				0.6			1.422154	1.4103210
					0.4		1.55721321	1.568231023
					0.6		1.56762421	1.57882314
						0.4	1.6320142	1.643102101
						0.6	1.7123210	1.72322372

**Table 2:** Nusselt number $Nu_{x} Re_{x}^{ - 0.5}$ versus various parameters for Blood-based hybrid nanofluid.

$\phi_{1} + \phi_{2}$	$\Lambda$	$M$	$Q$	$Ec$	$SWCNTs - Nu_{x}$	$SWCNTs + MWCNTs - Nu_{x}$
0.0	0.2	0.2	0.2	0.2	0.23414214	0.23414214
0.01					0.23625325	0.23956436
0.02					0.238436435	0.242675477
**0.03**					0.2401023532	0.245786587
	0.4				0.214120101	0.225221312
	0.6				0.2011071123	0.213219021
		0.4			0.3243123021	0.3354210312
		0.6			0.3363201293	0.34643120321
			0.4		0.34632115321	0.35743204310
			0.6		0.3539076321	0.36432109723
				0.4	0.41032034210	0.42370128301
				0.6	0.44532104320	0.45632107341

**Table 3:** Sherwood number versus the embedded parameters.

$\phi_{1} + \phi_{2}$	$Sc$	$\gamma$	$SWCNTs - Sh_{x}$	$SWCNTs + MWCNTs - Sh_{x}$
0.0	0.1	0.1	0.743308321	0.75321401
0.02			0.754219023	0.76320132
0.04			0.764320121	0.77543109
	0.3		0.823457012	0.83421089
	0.5		0.9310764351	0.94219082
		0.3	0.9457832101	0.956704231
		0.5	0.9533442156	0.962245713

## Conclusions

Health acquired infections (IACs) is a main public health issue worldwide. Whereas CNTs nanofluid plays its important role as antimicrobial. Carbon properties have a strong antimicrobial perspective and CNTs nanofluids are used in the Escherichia coli culture to assess their antibacterial potential. The improvement in the temperature distribution appraises the pH values and electric conductivity. Thus, the $SWCNTs\,\,{\text{and}}\,\,MWCNTs$ hybrid nanofluids are used in this study for medication purpose.

The core purpose of this research is to inspect the heat transfer and the effect of electro-magnetic field upon flow of a couple stress hybrid nanofluid over a Darcy-Forchheimer model in a symmetric flow with variable viscosity. Analytically, HAM is employed to solve the obtained set of equations in non-dimensional arrangement. The impact of encountered parameters has also discussed in detail. During this deep discussion, the underlined points have been revealed:

- In the presence of electric effects, the field of velocity decreases and at a certain distance away from the wall, it rises closed to the nonlinear stretching sheet.
- For increasing values of magnetic effect, there is a generation of Lorentz force in opposite direction of flow field that declines the velocity filed.
- For increasing values of porosity parameter the void spaces in the medium augments that offers more resistance to the flow of fluid and declines the flow profile.
- Augmentation in inertia coefficient results in generation of resistive force to fluid motion that causes a reduction in flow profile.
- For augmentation in Prandtl number the thermal diffusion reduces due to which less heat transfer takes place that ultimately declines the thermal profile.
- An increase in electric field accelerates the Lorentz force due to which temperature of nanoparticles increases.
- Augmentation in magnetic parameter corresponds to an augmentation in temperature profile.
- For higher values of Eckert number, the fluid friction amongst nanoparticles generates with more intensity. In this physical phenomenon the kinetic energy transformed to thermal energy that finally supports the augmentation in thermal profile.
- Augmenting values of volumetric friction causes increase in the dense behaviour of the fluid particles that depreciates the fluid flow and appreciates the thermal behaviour of fluid particles. Moreover, the concentration of fluid also rises in this phenomenon.
- An upsurge in Schmidt number results a reduction in concentration profile.
- Rising values in the chemical reaction parameter rapidly reduces the concentration profile.

## List of symbols

$$u\,{\text{and}}\,v$$: Velocity components
$$x\,{\text{and}}\,y$$: Cartesian coordinates
$T$: Hybrid nano fluid temperature
$b$: Stretching rate constant
$$T_{\infty }$$: Ambient temperature
$$M$$: Magnetic parameter
$$T_{w}$$: Wall temperature
$E$: Electric field parameter
$E_{0}$: Strength of electric field
$\rho_{hnf}$: Density of hybrid nanofluid
$\nu_{hnf}$: Hybrid nanofluid kinematic viscosity
$\alpha_{hnf}$: Hybrid nanofluid thermal diffusivity
$\left( {\rho c_{p} } \right)_{hnf}$: Hybrid nanofluid heat capacity
$$Ec$$: Eckert number
$$J$$: Current density
$C_{fx}$: Skin friction coefficient
$$\tau_{w}$$: Shear stress
$B_{0}$: Strength of magnetic field
$$\beta$$: Casson parameter
$$Nu_{x}$$: Nusselt number
$$Re_{x}$$: Reynolds number
$k = MLT^{ - 1}$: Couple stress parameter
$$Q_{0}$$: Heat flux
$$F^{\prime}$$: Dimensionless velocity
$$\infty$$: Ambient condition
$w$: Condition on surface
